# Community awareness, perception, and perceived behaviors regarding the impacts of advanced technology on the environment among residents of the eastern region, Saudi Arabia: a cross-sectional study

**DOI:** 10.3389/fpubh.2025.1649249

**Published:** 2025-10-01

**Authors:** Yousif M. Elmosaad, Ahmed M. Al Rajeh, Maria Blesilda B. Llaguno, Safia Belal, Bothaina H. Hassan, Mohammad Aatif, Ahmad Ibrahim, Munerah Almulhem, Humood Fahm Albugami, Abdel Moneim S. Elhassan, Eduardo L. Fabella, Heba M. Arakeep, Edwin C. Cancino, Edric D. Estrella, Sara Almaani, Abdullah S. Al hashem, Abdullah Ahmed Al Moweshy, Ghazi I. Al Jowf

**Affiliations:** ^1^Department of Public Health, College of Applied Medical Sciences, King Faisal University, Al Ahsa, Saudi Arabia; ^2^Department of Respiratory Care, College of Applied Medical Sciences, King Faisal University, Al Ahsa, Saudi Arabia; ^3^Department of Nursing, College of Applied Medical Sciences, King Faisal University, Al Ahsa, Saudi Arabia

**Keywords:** community awareness, behaviors, technology, environment, KSA

## Abstract

**Introduction:**

Technology is a major and indispensable part of everyone’s life, but the negative utilization of advanced technology has caused numerous global environmental problems, such as declining biodiversity, climate change, ozone depletion, overpopulation, and hazardous waste. The current study primarily aims to assess environmental awareness perceptions and perceived behaviors held by the community toward the Impacts of Advanced Technology on the Environment.

**Methods:**

An analytical cross-sectional study design was conducted among 310 residents of the three administrative areas of Al-Ahsa (Al-Hofuf, Al-Mubaraz, and Al-Gourah) in eastern Saudi Arabia from January to February 2024 with a response rate of 80.7%. A researcher-developed questionnaire consisting of four sections was utilized with a Cronbach’s alpha test result of (0.81). The data was analyzed using SPSS version 24, which included descriptive statistics, chi-square, and multivariate logistic regression. Crude and adjusted odds ratios were reported with corresponding 95% confidence interval estimates; statistical significance was set at an alpha less than 0.05.

**Results:**

More than half of the study participants exhibited a relatively good awareness level and a positive perception, especially males, younger respondents, those with bachelor’s degrees, and urban residents. Multivariate analysis showed that younger respondents with high education levels adjusted with good levels of awareness were more likely to develop positive perceptions toward the impacts of advanced technology on the environment. In addition, we observed that the participants with positive perceptions and good awareness were more likely to donate their equipment when not in use and use alternatives to technology. Awareness and positive perceptions motivate them to practice responsible behaviors toward the environment.

**Conclusion:**

There is a need for education and promotion programs to be implemented in the community to promote concern for the environment, encourage favorable perceptions to shape their practices and prepare them to continuously practice environmentally friendly activities.

## 1 Introduction

Technology is a major and indispensable part of everyone’s life at home, school, and work; there is approximately no place around the globe not using any technological devices due to rapid technological innovations like the production of more machines, weapons, and automobiles (1). The use of technology leads to environmental pollution due to industrialization, mismanagement, and lack of control measures which has greatly shaped and impacted society, the economy, and the environment (2, 3). Technologies are implicated in many contemporary harms, including waste production, depletion of resources and air, water, heat, and noise pollution (4). They have become principal threats to the ecosystem, public health, economic growth, and sustainable development (5, 6).

Numerous studies reported that the negative utilization of advanced technology caused numerous global environmental problems, such as declining biodiversity, climate change, ozone depletion, overpopulation, and hazardous wastes, thereby causing problems of air and water pollution and toxic waste disposal common in all industrialized countries (3, 7). Millions in developing countries lack access to sanitation services and safe drinking water, while dust and suspended materials contribute to hundreds of thousands of deaths yearly. Moreover, serious damage from pollution and overuse of renewable sources of energy challenges world fisheries, agriculture, and forests, with significant present and possible adverse effects on the physical environment (8).

The 21st century brought about significant environmental changes leading to global environmental problems, technological developments, and industrialization initiatives that revolutionized all aspects of human life (9). For example, in the United States, emissions of primary pollutants into the atmosphere are due to transportation (46%), fuel consumption in stationary sources (29%), industrial processes (16%), solid waste disposal (2%), and others (7%) (10). In the UK, the emissions of the basket of seven greenhouse gases covered by the Kyoto Protocol were estimated to be 451.5 million tons. Carbon dioxide (CO2) is the most dominant greenhouse gas (GHG), accounting for 81% of total UK greenhouse gas emissions (11). The consensus in the early 1990s was that the human-induced greenhouse effect had already warmed the earth by about 0.5 °C and a further warming of about 2.0 °C by 2030 (12). In this regard, Cassia et al. (13) identified in an experimental study that the short-term consequences of GHG increase in plants are mainly associated with the rise in atmospheric CO_2_. It likewise highlighted that in the absence of the greenhouse effect, the average temperature on the earth’s surface is estimated at around −19 °C instead of the current average of 14 °C (13). A survey of 33 countries showed that environmental issues did not rank first in any surveyed nations indicating that the world is soon entering an age of increased environmental apathy (14). Furthermore, technology contributes toward the depletion of resources by increasing industrial activity requiring raw materials from natural resources such as coal, timber, wild animals, forest cover, water, and soil fertility, and its organism’s composition is a likely event (15). On the other hand, technology plays a critical role as an instrument for observing and monitoring the environment on global and local scales and mitigating the effects of pollutants as well, as technology has been seen as the solution that arises from specifically driven research into practical problems (16). In addition, other studies suggested that the right employment of technology could save the environment.

Considering the previous discussions, humanity faces many environmental problems and challenges that adversely affect its health and well-being because it’s closely linked to the integrity of local, regional, and global ecosystems (17, 18). These problems cannot be managed only by cause-and-effect relationships, as they involve myriad factors and vary over time and space (16). Because of the interdependencies between humans and the environment, most environmental challenges require a fundamental increasing level of knowledge and changes in perceptions and behaviors among the population.

According to our knowledge, numerous studies have been conducted worldwide on technology and its impacts on the environment. While in Saudi Arabia, no studies, especially in Al-Ahsa, focused on the impacts of advanced technology on the environment and the role of community awareness in reducing the adverse impacts of technology on the environment and even human health. Therefore, we examined the status of environmental awareness and perceptions held by Al Ahsa residents toward the impacts of advanced technology on the environment. The study’s outcomes may support the delivery of information to authorities about public awareness and perceptions of the causes of the adverse impacts of technology on the environment. Thus, we hypothesized that the participants from different areas and with various demographic characteristics in the study area were asked about various environmental issues, and their awareness and perceived responses helped inform the planning and setting strategies for reducing adverse impacts of technology and making decisions regarding a sustainable environment.

## 2 Materials and methods

### 2.1 Study design and setting

This scholarly work employed an analytical cross-sectional study design among people living in the three administrative areas of Al-Ahsa (Al-Hofuf, Al-Mubaraz, and Al-Gourah) in the Eastern Region of Saudi Arabia. Based on voluntary and informed consent respondents filled out (written) the questionnaires during the period of Jan. 01, 2024 to Feb. 28, 2024. Data were fully anonymized to ensure that individual users could not be identified. The Ethical Clearance was obtained from the Deanship of Scientific Research at King Faisal University with reference number KFU-REC-2023-DEC-ETHICS1704.

### 2.2 Study population and sampling

The estimated population of the residents aged 18 years and above is (853072) individuals (Central Authority for Statistics, year 2022); 42.0% of them are residing in Al Hofuf, 38.0% in Al-Mubaraz, and 20%, in Al-Gourah. The minimum sample size of 381 was calculated using Epi Info® version 7 based on the following parameters; The estimated population of those aged 18 years and above is (853072) (19) (Al-Ahsa, Saudi Arabia – Population and Demographics https://www.city-facts.com/al-ahsa/population), confidence interval (CI) of 95%, anticipated frequency of 50% and maximum tolerable error of 5%. A total of 384 sample size was computed with a proportional allocation of the calculated sample size as follows: (160) for Al-Hofuf, (145) for Al-Mubaraz, and (76) for Al-Gourah. The sampling units for this study were drawn using a two-stage probability sampling procedure.

### 2.3 Instruments

The instrument used in this study was a researcher-developed questionnaire consisting of four sections. Section 1, comprised questions about the participants’ demographic characteristics, such as gender, age, education level, residence, and monthly income. Section 2, consisted of ten questions ascertaining the participants’ awareness of the impacts of advanced technology on the environment. Section 3, included two perceptional statements about the impacts of advanced technology on the environment; Section 4, consisted of two questions regarding respondents’ perceived behavior to reduce the negative impacts of advanced technology on the environment.

To test the reliability and validity of the questionnaire and to estimate the time required to answer all questions, the questionnaire was tested among (27) respondents who were selected randomly from study population. Pilot testing was also conducted to evaluate language, structure, understanding of the questions and duration. The reliability and validity of the tool was tested using Cronbach’s alpha test result of (0.81).

### 2.4 Variables

An overall score for the participant’s awareness was 10, calculated by adding the score for each question. The response to the questions was in the form of “Yes or No” and was given one point for each correct answer. The categorization of the participants was based on the mean score in each section and was dichotomized: participants who obtained a score equal to or higher than 6.5 points in the awareness section were considered as having a “good awareness level, while those with less than 6.5 were classified as having “poor awareness level.”

The perception questions’ responses were a Likert-type scale with four dimensions, “strongly agree, agree, disagree, and strongly disagree,” with a maximum score of (8).

Four points were allotted for strongly agree and one point, for strongly disagree responses. Similarly, the perception scores were dichotomized: those with a score equal to or higher than 6.0 points were considered to have positive perception, while those with a score of less than 6.0 points possessed negative perception.

The responses to questions pertaining on perceived behavior were also in the form of “Yes or No,” with one point for each correct answer considered to be having a good practice and zero point for an incorrect answer considered to be having poor practice.

### 2.5 Data collection

The data was collected from the first week of January to the last week of February 2024. The research tool was administered to possible participants through a survey tool provided with English and Arabic translations.

### 2.6 Data analysis

All collected data were encoded, processed in Microsoft Excel, and analyzed using SPSS (Version 24). Summary statistics were presented for the demographic characteristics of the respondents. The mean, standard deviation, and highest and lowest scores were calculated for continuous variables such as age, awareness level, and perceptions. Awareness level, perception, and perceived behavior proportions were estimated. Moreover, the Chi-square test was utilized to determine the association between the participants’ socio-demographic variables, awareness level, and perceptions of the impacts of advanced technology on the environment. Additionally, multivariate logistic regression was performed to adjust for the confounding effects of age group, gender, residence, and monthly income. Crude and adjusted odds ratios were reported with corresponding 95% confidence interval estimates. Statistical significance was set at an alpha less than 0.05.

## 3 Results

### 3.1 Socio-demographic characteristics of the study participants

Table 1 summarizes the socio-demographic characteristics of the study participants. A total of 310 residents participated in the study, 52.3% from Al-Hofuf, 32.2% from Al-Garah, and 24.5% from Al-Mubaraz. The males exceeded the female participants, with (61.6%) and (38.4%), respectively. As with their educational level, (35.2%) were bachelor’s degree holders, followed by high school then by lower degree holders (28.7%). There were (19.7%) college graduates, (13.5%) university students and a small proportion (2.9%) were at the postgraduate level. Most of the participants were youngsters (68.1%), and older people (16.5%), while middle-aged individuals have the least proportion (15.5%), with a mean age of 27.2 ± 8.6 years. More than half of them (50.5%) have a high level of income (>15,000 SAR), followed by those in the low bracket (22.9%) and only (9.7%) from the middle level of income, respectively.

**Table 1:** Socio-demographic characteristics of the study participants (*n* = 310).

Variables	No. of respondents	Proportion (in %)
Gender		
Male	191	61.6
Female	119	38.4
Age group		
18–27 years	211	68.1
28–37 years	48	15.5
>37 years	51	16.5
Education level		
High school and lower	89	28.7
College diploma	61	19.7
Bachelor’s degree	109	35.2
University students	42	13.5
Postgraduate (Master/PhD)	9	2.9
Residence		
Al-Hofuf	162	52.3
Al-Mubaraz	76	24.5
Al-Garah	72	32.2
Monthly income		
4,000–9,000 SAR	64	22.9
9,001–15,000 SAR	27	9.7
>15,000 SAR	141	50.5

Mean age of the respondents = (27.2 ± 8.6) years.

### 3.2 Participants’ awareness of the impacts of advanced technology on the environment

An overall score of participant’s awareness was calculated by adding the score for each of all ten questions, and the maximum score for the awareness variable was 10 (The response to the questions was in the form of Yes or No and one point for each correct answer). An awareness score of 6.5 or above was considered a good awareness level.

A set of statements were developed to measure the participants’ awareness of the impacts of advanced technology on the environment. As shown in Table 2, more than half of the study participants (51.9%) showed good awareness of the impacts of advanced technology on the environment. As a result, approximately 91.3% of the study participants knew the importance of maintaining a continuous link between technology and human behavior to protect the environment, 83.9% of them agreed that technology is a double-edged sword, 83.5% knew that the impact of technology on environment is not uniform throughout the world, and 76.1% agreed that the impacts of technology on the environment depend on what and how technologies are used. Moreover, 75.5% knew that waste disposal generated by technology without scientific measures could cause environmental pollution. However, it is noteworthy that more than 60% of the study participants agreed that utilizing renewable energy sources and reusing older devices could help reduce technology’s negative effects on the environment. In comparison, 64.8% did not blame technology as a major contributor to the pollution that contributes to global warming.

**Table 2:** Participants’ awareness of the negative impacts of advanced technology on the environment.

Awareness variables	Correct responses	
	Frequency	Percent
The use of the technology leads to environmental degradation. (Yes)	102	32.9
Technology is a double-edged sword. (Yes)	260	83.9
Technology produces solid, liquid, and gaseous wastes that negatively affect the environment. (Yes)	165	53.2
Technology is to be blamed for much of the pollution that contributes to global warming. (Yes)	109	35.2
Disposal of waste generated by technology without scientific measures can cause environmental pollution. (Yes)	234	75.5
Reusing older devices is one of the things that we can do to reduce the negative effect of technology on the environment. (Yes)	189	61.0
The impacts of technology on the environment depend on what and how technologies are used. (Yes)	236	76.1
Utilizing renewable energy sources in Saudi Arabia can help to reduce the negative effects of technology on the environment. (Yes)	188	60.6
Maintaining a continuous link between technology and human behavior is important to protect the environment. (Yes)	283	91.3
The impact of technology on the environment is not uniform throughout the world. (Yes)	259	83.5
Overall awareness level		
Poor (less than average mean 6.5)	149	48.1
Good (more than average mean 6.5)	161	51.9

An overall score of the participant’s awareness was calculated by adding up the score for each of the 10 questions, and the maximum score for the ‘awareness’ variable was 10 (the response to the questions was in the form of Yes or No and one point for each correct answer). An awareness score of 6.5 or above was considered as a good awareness level.

### 3.3 Participants’ perceptions of the impacts of advanced technology on the environment

An overall score of the participant’s perception was calculated by adding up the score for each of the questions, and the maximum score for the ‘perception’ variable was 8 [The response to the questions was in the form of a Likert-type scale with four dimensions (strongly agree, agree, disagree, and strongly disagree)]. A perception score of 6.5 or above was considered a positive perception (68.1%).

**Figure 1:**
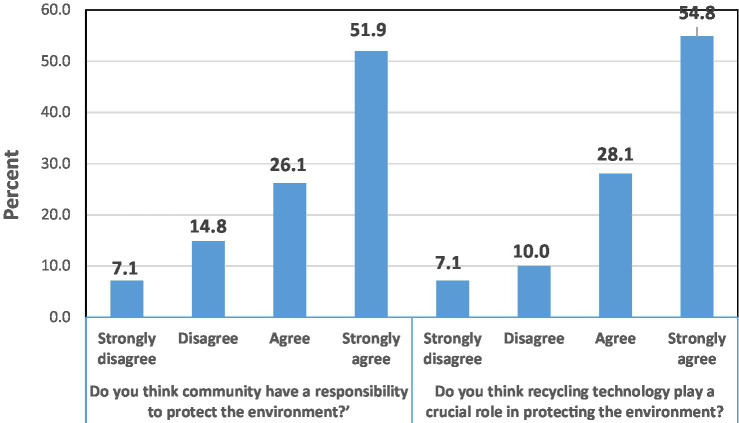
Participants’ perceptions of the impacts of advanced technology on the environment.

It can be seen from Figure 1 that 68.1% of study participants have a positive perception of the impacts of advanced technology on the environment. The mean (±SD) score for perceptions was (6.5 ± 1.4) with a range of two to eight (2–8). Most of the study participants agreed that the community protects the environment. Moreover, 82.9% of them agreed that recycling technology plays a crucial role in protecting the environment (See Figure 1). This may suggest that while people may have positive perceptions toward the impacts of advanced technology on the environment, they may also desire to use other technologies that are environment-friendly.

### 3.4 Association between the participants’ socio-demographic variables, awareness level, and perceptions toward the impacts of advanced technology on the environment

Table 3 shows that the Chi-square test result indicates that some socio-demographic characteristics were significantly associated with the respondents’ awareness level about the impacts of advanced technology on the environment (*p* < 0.05). These were gender, age, education, and residence. Specifically, the males (58.6%), younger population from 18 to 27 years old (56.4%), those with bachelor’s degrees (58.7%), and residents of Al-Hofuf (58.0%) have a high level of awareness. On the other hand, the results showed that the awareness level was not associated with monthly income (*p* > 0.05).

**Table 3:** Association between the participants’ socio-demographic variables, awareness level, and perceptions toward the impacts of advanced technology on the environment.

Variables	Awareness level		Sig.	Overall perceptions		Sig.
	Poor (No./%)	Good (No./%)		Negative (No./%)	Positive (No./%)	
Gender						
Male	79 (41.4)	112 (58.6)	0.002	53 (27.7)	138 (72.3)	0.031
Female	70 (58.8)	49 (41.2)		46 (38.7)	73 (61.3)	
Age group						
18–27 years	92 (43.6)	119 (56.4)	0.013	59 (28.0)	152 (72.0)	0.027
28–37 years	23 (47.9)	25 (52.1)		23 (47.9)	25 (52.1)	
>37 years	34 (66.7)	17 (33.3)		17 (33.3)	34 (66.7)	
Education level						
High school and lower	47 (52.8)	42 (47.2)	0.049	32 (36.0)	57 (64.0)	0.016
College diploma	37 (60.7)	24 (39.3)		17 (27.9)	44 (72.1)	
Bachelor’s degree	45 (41.3)	64 (58.7)		25 (22.9)	84 (77.1)	
University students	18 (42.9)	24 (57.1)		21 (50.0)	21 (50.0)	
Postgraduate (Master/PhD)	2 (22.2)	7 (77.8)		4 (44.4)	5 (55.6)	
Residence						
Al-Hofuf	68 (42.0)	94 (58.0)	0.004	51 (31.5)	111 (68.5)	0.977
Al-Mubaraz	34 (44.7)	42 (55.3)		25 (32.9)	51 (67.1)	
Al-Garah	47 (65.3)	25 (34.7)		23 (31.9)	49 (68.1)	
Monthly income						
4,000–9,000 SAR	99 (50.5)	97 (49.5)	0.301	61 (31.1)	135 (68.9)	0.562
9,001–15,000 SAR	36 (47.4)	40 (52.6)		23 (30.3)	53 (69.7)	
>15,000 SAR	149 (48.1)	161 (51.9)		15 (39.5)	23 (60.5)	

Regarding the study participants’ perceptions of the impacts of advanced technology on the environment. The results showed a significant association with gender, age group, and educational level (*p* < 0.05). However, males (72.3%), the younger population (72.0%), and the bachelor’s degree holders (77.1%) were associated with positive perceptions, whereas residence and average monthly income were not associated (*p* > 0.05).

### 3.5 Predictors of participants’ perceptions toward the impacts of advanced technology on the environment adjusted by awareness level

Multivariate analysis showed that some socio-demographic variables - age and education level - were significantly associated with study participants’ perceptions of the impacts of advanced technology on the environment (*p* < 0.05). Specifically, those aged (28–37) and (>37) years old were found to be less likely to have a positive perception [*B* = −1.18 and −0.23] respectively, than the younger study participants (18–27 years old). Even though all participants have different socio-demographic variables and they have good awareness levels, they were more likely to develop positive perceptions toward the impacts of advanced technology on the environment than those who have poor awareness levels (*B* = 1.65, OR = 5.20, 95% CI: 2.86–9.45). However, gender, residence, and average monthly income were not associated with the study participant’s perceptions of advanced technology’s negative impacts on the environment (*p* > 0.05). Thus, we can conclude that changing and developing perceptions is very complicated, as awareness alone is not enough, despite having a strong influence. However, people have other motivational factors that affect the complex interaction between awareness, demographic factors, and the development of perceptions (Table 4).

**Table 4:** Predictors of participants’ perceptions toward the impacts of advanced technology on the environment adjusted by awareness level.

Predictors	Perceptions		B	Unadjusted model		B	Adjusted model	
	Negative (No./%)	Positive (No./%)		OR (95% CI)	*p*-value		OR (95% CI)	*p*-value
Awareness level								
Poor	70 (22.6)	79 (25.5)	Ref.	–	–	–	–	–
Good	29 (9.4)	132 (42.6)	1.40	4.03 (2.41–6.75)	0.001	1.65	5.20 (2.86–9.45)	0.0001
Gender								
Male	53 (17.1)	138 (44.5)	Ref.	–	–	–	–	–
Female	46 (14.8)	73 (23.5)	−0.49	0.61 (0.37–0.99)	0.046	−0.38	0.69 (0.39–1.22)	0.198
Age group								
18–27 years	59 (19.0)	152 (49.0)	Ref.	–	–	–	–	–
28–37 years	23 (7.4)	25 (8.1)	−0.863	0.42 (0.22–0.80)	0.008	−1.18	0.31 (0.14–0.66)	0.002
>37 years	17 (5.5)	34 (11.0)	−0.253	0.78 (0.40–1.50)	0.449	−0.23	0.79 (0.38–1.67)	0.542
Education level								
High school and lower	32 (10.3)	57 (18.4)	Ref	–	–	–	–	–
College diploma	17 (5.5)	44 (14.2)	0.37	1.45 (0.72–2.95)	0.301	0.50	1.65 (0.74–3.65)	0.218
Bachelor degree	25 (8.1)	84 (27.1)	0.64	1.89 (1.01–3.51)	0.046	0.59	1.80 (0.91–3.59)	0.093
University students	21 (6.8)	21 (6.8)	−0.35	0.70 (0.18–2.80)	0.616	−1.07	0.71 (0.15–3.33)	0.662
Postgraduate (Master/PhD)	4 (1.3)	5 (1.6)	−0.58	0.56 (0.27–1.18)	0.128	−0.35	0.34 (0.15–0.81)	0.015
Residence								
Al-Hofuf	51 (16.5)	111 (35.8)	Ref	–	–	–	–	–
Al-Mubaraz	25 (8.1)	51 (16.5)	0.021	1.02 (0.56–1.85)	0.944	0.06	1.06 (0.54–2.09)	0.859
Al-Garah	23 (7.4)	49 (15.8)	−0.043	0.96 (0.48–1.91)	0.902	0.61	1.84 (0.90–3.77)	0.094
Monthly income								
4,000–9,000 SAR	61 (19.7)	135 (43.5)	Ref	–	–	–	–	–
9,001–15,000 SAR	23 (7.4)	53 (17.1)	0.04	1.04 (0.59–1.85)	0.891	0.02	1.02 (0.54–1.94)	0.947
>15,000 SAR	15 (4.8)	23 (7.4)	−0.37	0.69 (0.34–1.42)	0.316	−0.45	0.64 (0.27–1.52)	0.311

Dependent variable: types of perceptions about the negative effect of advance technology on the environment.

### 3.6 Perceived behavior to reduce the negative impacts of advanced technology on the environment

**Figure 2:**
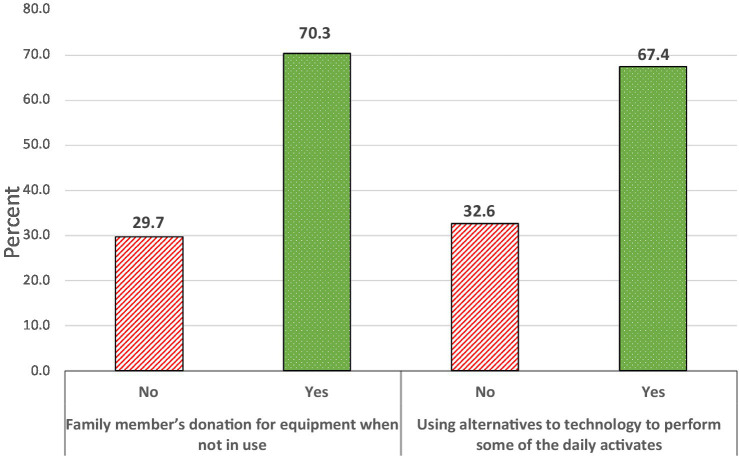
Perceived behavior to reduce the negative impacts of advanced technology on the environment.

Regarding the perceived behavior to reduce the negative impacts of advanced technology on the environment, Figure 2 shows that more than two-thirds of study participants use alternative technology in their daily activities. Moreover, 70.3% of study participants donated their equipment when they could not use it to reduce advanced technology’s negative impacts on the environment. These findings showed that the study participants believe in the positive outcomes associated with donating unused equipment and using alternative technology on the environment. Positive outcomes act as a significant stimulus for individuals to integrate environmental-friendly practices in their daily activities on various environmental issues. This indicates that assessing and understanding perceived behavior lead to promote healthier lifestyles, influence social norms and motivate community members to engage in sustainable behavior, which is reflected in practicing environmentally friendly behavior.

### 3.7 Predictors on perceived behavior to reduce the negative impacts of advanced technology on the environment adjusted by socio-demographic variables

We observed that participants with positive perceptions, good awareness, and high monthly income were more likely to donate their equipment when not in use (*β* = 1.19, 0.79, 0.98), respectively. However, no significant differences in the odds of equipment donation were observed between other socio-demographic variables. It also showed that the odds of using alternatives to technology to reduce the negative impacts of advanced technology on the environment was (OR = 2.79, 1.81) times of using alternatives to technology among participants with positive perception and good awareness levels, respectively. In contrast, females were significantly less likely to use alternatives to technology (*β* = − 0.61). However, no significant differences in the odds of using alternatives to technology were observed between other socio-demographic variables (Table 5).

**Table 5:** Predictors on perceived behavior to reduce the negative impacts of advanced technology on the environment adjusted by socio-demographic variables.

Predictors	Perceived behavior to reduce the negative impacts of advanced technology on the environment									
	Family member’s donation for equipment when not in use					Using alternatives to technology				
	No (No./%)	Yes (No./%)	Adjusted model			No (No./%)	Yes (No./%)	Adjusted model		
			B	OR (95% CI)	*p*-value			B	OR (95% CI)	*p*-value
Perceptions										
Negative	49 (15.8)	43 (13.9)	Ref.	–	–	50 (16.1)	49 (15.8)		–	–
Positive	50 (16.1)	168 (54.2)	1.19	3.29 (1.84–5.90)	0.000	51 (16.5)	160 (51.6)	1.03	2.79 (1.56–5.01)	0.001
Awareness level										
Poor	59 (19.0)	33 (10.6)	Ref.	–	–	67 (21.6)	82 (26.5)		–	–
Good	90 (29.0)	128 (41.3)	0.79	2.21 (1.22–4.00)	0.009	34 (11.0)	127 (41.0)	0.59	1.81 (1.03–3.18)	0.040
Gender										
Male	54 (17.4)	137 (44.2)	Ref.			50 (16.1)	141 (45.5)		–	–
Female	38 (12.3)	81 (26.1)	0.04	1.04 (058–1.85)	0.900	51 (16.5)	68 (21.9)	−0.61	0.54 (0.31–0.95)	0.033
Age group										
18–27 years	64 (20.6)	147 (47.4)	Ref.	–	–	59 (19.0)	152 (49.0)		–	–
28–37 years	12 (3.9)	36 (11.6)	0.52	1.68 (0.73–3.87)	0.226	18 (5.8)	30 (9.7)	−0.32	0.73 (0.34–1.56)	0.412
>37 years	16 (5.2)	35 (11.3)	0.03	1.03 (0.48–2.20)	0.328	24 (7.7)	27 (8.7)	−0.72	0.48 (0.24–0.99)	0.048
Education level										
High school and lower	25 (8.1)	64 (20.6)	Ref.	–	–	30 (9.7)	59 (19.0)	–	–	–
College diploma	17 (5.5)	44 (14.2)	−0.02	0.98 (0.44–2.18)	0.217	20 (5.6)	41 (13.2)	−0.21	0.81 (0.38–1.76)	0.600
Bachelor degree	29 (9.4)	80 (25.8)	−0.22	0.80 (0.401.59)	0.283	35 (11.3)	74 (23.9)	−0.28	0.76 (0.39–1.47)	0.412
University students	18 (5.8)	24 (7.7)	−0.65	0.52 (0.09–2.87)	0.458	14 (4.5)	28 (9.0)	1.06	2.90 (0.42–9.91)	0.280
Postgraduate (Master/PhD)	3 (1.0)	6 (1.9)	−0.55	0.57 (0.24–1.38)	0.918	2 (0.6)	7 (2.3)	−0.22	0.80 (0.33–1.95)	0.631
Residence										
Al-Hofuf	46 (14.8)	116 (37.4)	Ref.	–	–	47 (15.2)	115 (37.1)	–	–	–
Al-Mubaraz	26 (8.4)	50 (16.1)	−0.25	0.79 (0.40–1.52)	0.467	20 (6.5)	56 (18.1)	0.36	1.44 (0.73–2.85)	0.296
Al-Gara	20 (6.5)	52 (16.8)	−0.48	1.27 (0.61–2.62)	0.523	34 (11.0)	38 (12.3)	−0.57	0.56 (0.29–1.10)	0.094
Monthly income										
4,000–9,000 SAR	65 (21.0)	131 (42.3)	Ref.	–	–	68 (21.9)	128 (41.3)	–	–	0.320
9,001–15,000 SAR	13 (4.2)	63 (20.3)	0.98	2.66 (1.30–5.42)	0.007	22 (7.1)	54 (17.4)	0.47	1.60 (0.84–3.05)	0.150
>15,000 SAR	14 (4.5)	24 (7.7)	−0.02	0.98 (0.43–2.22)	0.963	11 (3.5)	27 (8.7)	0.31	1.37 (0.58–3.22)	0.471

Dependent variable: Perceived behavior to reduce the negative effect of advance technology on the environment.

## 4 Discussion

The United Nations announced that advanced technology utilization could create a complex web of unforeseen negative environmental consequences currently endangering many countries due to a lack of environmentally friendly awareness and irresponsible environmental behavior (20, 21). In Saudi Arabia, industrial growth, rapid urbanization, and a high-consumption lifestyle have led to the utilization of advanced technologies, which may create many environmental issues. In response to these issues, government policies recently focused on raising awareness and strengthening individual and collective feelings of responsibility for preserving and improving the environment. Previous studies confirmed that when people have environmental awareness, they gain an environmentally friendly perception that motivates them to responsible behaviors to care for the environment (22, 23). In this context, assessing people’s awareness, perception, and behavior is essential to create better environmental protection policies to reduce the adverse effects of advanced technology on the environment. The overall results of this study showed that more than half of the study participants exhibited a relatively good awareness level about the impacts of the advanced technology on the environment. This result is consistent with the findings of the study conducted in Bangladesh, revealing that the respondents had a moderate level of perceived knowledge about the causes of environmental pollution (23). Likewise, another study conducted in India among Higher Primary School Teachers showed that most of them had moderate levels of environmental awareness (24). The studies conducted in Oman (25), Malaysia (26), and Bangladesh (27) have reported that most respondents were aware of the environment. As with the respondents’ level of awareness, it was reported that the residents have unsatisfactory despite the government’s dedicated great efforts through environmental laws and regulations focusing on raising environmental awareness and strengthening individual and collective feelings of responsibility for preserving and improving the environment (28). These endeavors reflect that other motivational factors may influence the public’s level of environmental awareness.

Regarding the respondents’ perceptions of the impacts of advanced technology on the environment, our results indicated that two-thirds of study participants have a positive perception reflected in their agreement about the community and that recycling technology plays a crucial role in protecting the environment. These results were partially consistent with a study done in Puerto Rico which reported that government agencies and communities are responsible for responding to environmental risks related to the use of technology (29). In terms of recycling technology, a study in Malaysia showed that waste recycling technology significantly decreases ecological footprints (26). Despite these findings, people desire to use technology but must consider environmental considerations.

Our finding showed that some socio-demographic characteristics were significantly associated with the respondents’ awareness level of the adverse impacts of advanced technology on the environment. Specifically, males, younger respondents, those with bachelor’s degrees, and urban residents have a high level of awareness. These findings are consistent with the studies conducted in developing countries such as China (30) and Malaysia (31). Concerning the association between gender and environmental awareness, many studies that have explored this association remain controversial. For example, studies conducted in Oman, India, and Malaysia have shown that females have significantly higher levels of environmental awareness than their male counterparts (24, 25, 31). In contrast, a study conducted in Bangladesh reported that both males and females were shown to have lower levels of environmental awareness (3). However another study conducted among secondary school students stated that males were more knowable than females about environmental issues (32).

Concerning the respondents’ perceptions of the impacts of advanced technology on the environment, our results showed that being male, younger, and bachelor’s degree holders were significantly associated with positive perceptions toward the use of technologies that are environment-friendly. These findings are consistent with studies conducted in developing countries such as Turkey (33), Bangladesh (3), Kenya (34), and Malaysia (31). Many studies have explored the association between socio-demographic characteristics and perceptions, and this association remains controversial. For instance, some studies reported that having a higher education degree is associated with poor perceptions (34, 35). In contrast, other studies indicate that a low education level significantly determines annoyance with perceived air pollution levels (36). Despite the controversial association of demographic variables with awareness and perceptions of the adverse effects of technology on the environment, they remain essential in environmental research because they represent underlying aspects that affect susceptibility and exposure to environmental factors. Therefore, there is a need for careful selection and interpretation of social indicators depending on their relevance to the problem under study.

Multivariate analysis showed that younger individuals with high education degrees adjusted with a good level of awareness were more likely to develop positive perceptions toward the impacts of advanced technology on the environment. Thus, developing and changing perceptions is complicated, as more than awareness is required. Despite having a significant influence, people may have other motivational factors that affect the complex interaction between awareness, demographic factors, and the development or changing of perceptions.

Regarding the perceived behavior to reduce the adverse impacts of advanced technology on the environment. Our finding shows that more than two-thirds of study participants use alternative technology in their daily activities and donate their equipment when not in use. Regarding alternative technology in literature, it has been explained to ensure a secure supply of environmentally friendly technology that avoids potential future materials crises (37–40). On the other hand, the literature also explains equipment donation as an activity that conserves natural resources and avoids air and water pollution caused by manufacturing virgin materials (36, 41, 42). Based on the previous discussion, our community knows these activities benefit in reducing adverse environmental consequences. Thus, there is a need for a promotion program to prepare them to practice these environmentally friendly and other activities continually.

Concerning the predictors on perceived behavior to reduce the negative impacts of advanced technology on the environment adjusted by socio-demographic variables. It can be noted that the participants who had positive perceptions and good awareness were more likely to donate their equipment when not in use and using alternatives to technology. Several studies have explored the value of perception and awareness in shaping individual behavior (22, 26, 43–45). It emphasized that when individuals possess the awareness and positive perceptions, these motivate them to practice responsible behavior toward the environment, such as donating their equipment when not in use, using alternatives to technology, and other activities with significant environmental effects. These findings reveal the value of awareness programs among the community to build environmental concern and encourage positive perceptions to shape their practices toward the environment. This suggestion supports (46), who reported that environmental education is the foundation for creating an environmentally conscious society and a more ethical community (45). The main limitation of this study is that data was gathered from the study participants using a survey and self-reported data; therefore, we cannot avoid the possibility of information bias, and a cross-sectional study cannot establish cause-and-effect relationships. Moreover, a lower response rate was obtained from the Al-Mubaraz area. However, these limitations may affect the generalizability of the study results.

## 5 Conclusion

In conclusion, this study has given supportive evidence to stockholders and academics regarding the association between and among the community’s awareness, perceptions, and behaviors regarding the undesirable effects of advanced technology on the environment. We found that more than half of the study participants exhibited a relatively good awareness level and a positive perception, especially males, younger respondents, those with bachelor’s degrees, and urban residents. Multivariate analysis showed that younger respondents with high education levels adjusted with good levels of awareness were more likely to develop positive perceptions toward the negative impacts of advanced technology on the environment. In addition, we observed that the participants with positive perceptions and good awareness were more likely to donate their equipment when not in use and use alternatives to technology. Awareness and positive perceptions motivate them to practice responsible behaviors toward the environment. In this regard, there is a need for education and promotion programs to be implemented in the community to promote concern for the environment, encourage favorable perceptions to shape their practices and prepare them to practice these environmentally friendly and other desirable activities continuously. In addition, researchers should carefully select and interpret social indicators depending on their relevance to the problem under study.

## Data Availability

The raw data supporting the conclusions of this article will be made available by the authors, without undue reservation.
